# Untypically mild phenotype of a patient suffering from Sanfilippo syndrome B with the c.638C>T/c.889C>T (p.Pro213Leu/p.Arg297Ter) mutations in the *NAGLU* gene

**DOI:** 10.1002/mgg3.1356

**Published:** 2020-06-24

**Authors:** Karolina Pierzynowska, Arkadiusz Mański, Monika Limanówka, Jolanta Wierzba, Lidia Gaffke, Paulina Anikiej, Grzegorz Węgrzyn

**Affiliations:** ^1^ Department of Molecular Biology Faculty of Biology University of Gdańsk Gdansk Poland; ^2^ Psychological Counselling Centre of Rare Genetic Diseases University of Gdańsk Gdansk Poland; ^3^ Departement of Pediatrics, Hematology and Oncology Medical University of Gdańsk Gdansk Poland; ^4^ Department of Internal and Pediatric Nursing Medical University of Gdańsk Gdansk Poland

**Keywords:** genotype, mucopolysaccharidosis, phenotype, Sanfilippo syndrome type B

## Abstract

**Background:**

Sanfilippo syndrome B (or mucopolysaccharidosis type IIIB [MPS IIIB]) is a severe inherited metabolic disorder caused by mutations in the *NAGLU* gene, encoding α‐N‐acetylglucosaminidase. Dysfunction of this enzyme results in impaired degradation of heparan sulfate, one of glycosaminoglycans, and accumulation of this complex carbohydrate in lysosomes. Severe symptoms occurring in this disease are related to progressive neurodegeneration and include extreme hyperactivity, sleeping problems, aggressive‐like behavior, reduced fear, and progressive mental and cognitive deterioration. No cure is currently available for Sanfilippo disease.

**Methods:**

Clinical characterization of the patient's symptoms has been performed. Biochemical analyses included glycosaminoglycan level determination and measurement of α‐N‐acetylglucosaminidase activity. Molecular analyses included exome sequencing and detailed analysis of the *NAGLU* gene. Psychological tests included assessment of attention, communication and behavior.

**Results:**

We describe a patient with an untypically mild phenotype, who was diagnosed at the age of 13 years. Many cognitive, communication, and motoric functions were preserved in this patient, contrary to vast majority of those suffering from MPS IIIB. The patient is a compound heterozygote (c.638C>T/c.889C>T) in the *NAGLU* gene, and relatively high residual activity (about 25%) of α‐N‐acetylglucosaminidase was measured in serum (while no activity of this enzyme could be detected in dry blood spot).

**Conclusions:**

We suggest that the mild phenotype might arise from the partially preserved function of the mutant enzyme (p.Pro213Leu), suggesting the genotype‐phenotype correlation in this case.

## Graphical abstract

Patient ZK, who was diagnosed for Sanfilippo syndrome B, is a compound heterozygote (c.638C>T/c.889C>T) in the NAGLU gene, with a relatively high (about 25%) residual activity of α‐N‐acetylglucosaminidase. She expressed relatively mild phenotype, with various cognitive and communication functions preserved, and relatively good motoric functions, at the age of 13 years. We suggest that this mild phenotype might arise from partially preserved activity of the p.Pro213Leu variant of α‐N‐acetylglucosaminidase, causing slow accumulation of HS and slow progress of the disease.

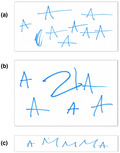

## 1 INTRODUCTION

Mucopolysaccharidosis (MPS) is a group of disorders belonging to lysosomal storage diseases (LSD) in which accumulation of undegraded glycosaminoglycans (GAGs) is the primary cause of dysfunctions of cells, organs and whole organisms (Tomatsu et al., 2018). Sanfilippo disease or mucopolysaccharidosis type III (MPS III) consists of four disorders in which heparan sulfate (HS) is the primary stored GAG due to deficiency in activity of one of following enzymes involved in degradation of this complex carbohydrate: N‐sulfoglucosamine sulfhydrolase (EC 3.10.1.1, the *SGSH* gene product), α‐N‐acetylglucosaminidase (EC 3.2.1.50, the *NAGLU* gene product), Acetyl‐CoA:α‐glucosaminide acetyltransferase (EC 2.3.1.78, the *HGSNAT* gene product) or N‐acetylglucosamine‐6‐sulfatase (EC 3.1.6.14, the *GNS* gene product), while secondary mild storage of dermatan sulfate (DS) has also been reported in this disease (Lamanna, Lawrence, Sarrazin, & Esko, 2011). These four disorders are named subtypes A (OMIM: 252900), B (OMIM: 252920), C (OMIM: 252930) and D (OMIM: 252940) of Sanfilippo disease, respectively. Although degradation of HS stops at various stages in each subtype, symptoms observed in patients suffering from MPS III are similar (for a review, see Jakóbkiewicz‐Banecka et al., 2016).

Contrary to other MPS types, Sanfilippo disease is characterized with relatively mild somatic symptoms while dysfunctions of central nervous system are particularly severe. MPS III patients are usually extremely hyperactive, sleeping problems are very serious, aggressive‐like behavior is common as is reduced fear, and progressive mental and cognitive deterioration is evident. Vast majority of patients cannot speak, and even if they develop speech to some extent, it is gradually lost in the course of the disease. Average life span is estimated to be about two decades (for a review, see Jakóbkiewicz‐Banecka et al., 2016; Tomatsu et al., 2018). Accumulation of HS in the brain tissues, accompanied by neuroinflammation, oxidative stress, and disturbances in many cellular processes, are believed to be responsible for these symptoms (Heon‐Roberts, Nguyen, & Pshezhetsky, 2020; Węgrzyn et al., 2010), though recent transcriptomic studies suggested that secondary or tertiary changes in expression of various behavior‐related genes may significantly contribute to development of cognitive and behavioral changes in Sanfilippo disease (Pierzynowska, Gaffke, Podlacha, & Węgrzyn, 2020).

Despite occurrence of a generally common picture of symptoms occurring in MPS III patients, their severity in individual children may vary significantly. The age of diagnosis can also be very different (from typical diagnosis at 4 years to cases diagnosed in adulthood); in fact, MPS III children sometimes develop symptoms resembling idiopathic developmental delay, attention deficit/hyperactivity disorder or autism spectrum disorders, often causing misdiagnosis (Wijburg, Węgrzyn, Burton, & Tylki‐Szymańska, 2013). Although currently there is no cure for Sanfilippo disease, many different therapeutic options are being investigated, and proper diagnosis made as rapidly as possible, accompanied by subsequent appropriate care, is extremely important for MPS III patients and their families (Gaffke, Pierzynowska, Piotrowska, & Węgrzyn, 2018). Therefore, description of cases of untypical MPS III patients may be crucial to understand details of pathomechanisms and variability of the course of the disease which should lead to better management of this severe disease in various patients. Here, we describe an untypically mild phenotype of one Sanfilippo syndrome B patient, providing results of clinical, molecular, and psychological investigations, which should contribute to our understanding of variability of the course of this disease.

## 2 CASE DESCRIPTION: CLINICAL AND MOLECULAR ANALYSES

Patient Z has been diagnosed for Sanfilippo syndrome B at the age of 13 years, after molecular investigations (see below). The family history demonstrated healthy, non‐consanguineous parents. There was no family history for mental retardation or neuropsychiatric diseases.

The girl was born after high risk pregnancy (light bleeding in the first trimester) at term; birth weight was 3,600 g (50 percentile), head circumference was 36 cm (95 percentile), Apgar score was 10, and no neonatal problems have been reported. Early psychomotor development was within normal limits, sitting position was achieved by the 8th month, autonomous walking by the 13th month of life. She was breastfed for 9 months with no sucking problems. During first 2 years of life, mild diffuse hypertrichosis was observed. Verbal communication has developed with simple sentences. The speech therapy was introduced in third year because of slurred speech. In the clinical history, no significant diseases were reported except from the recurrent common upper respiratory tract infections during first years of life. No adenotonsillar hypertrophy was diagnosed.

**Figure 1:**
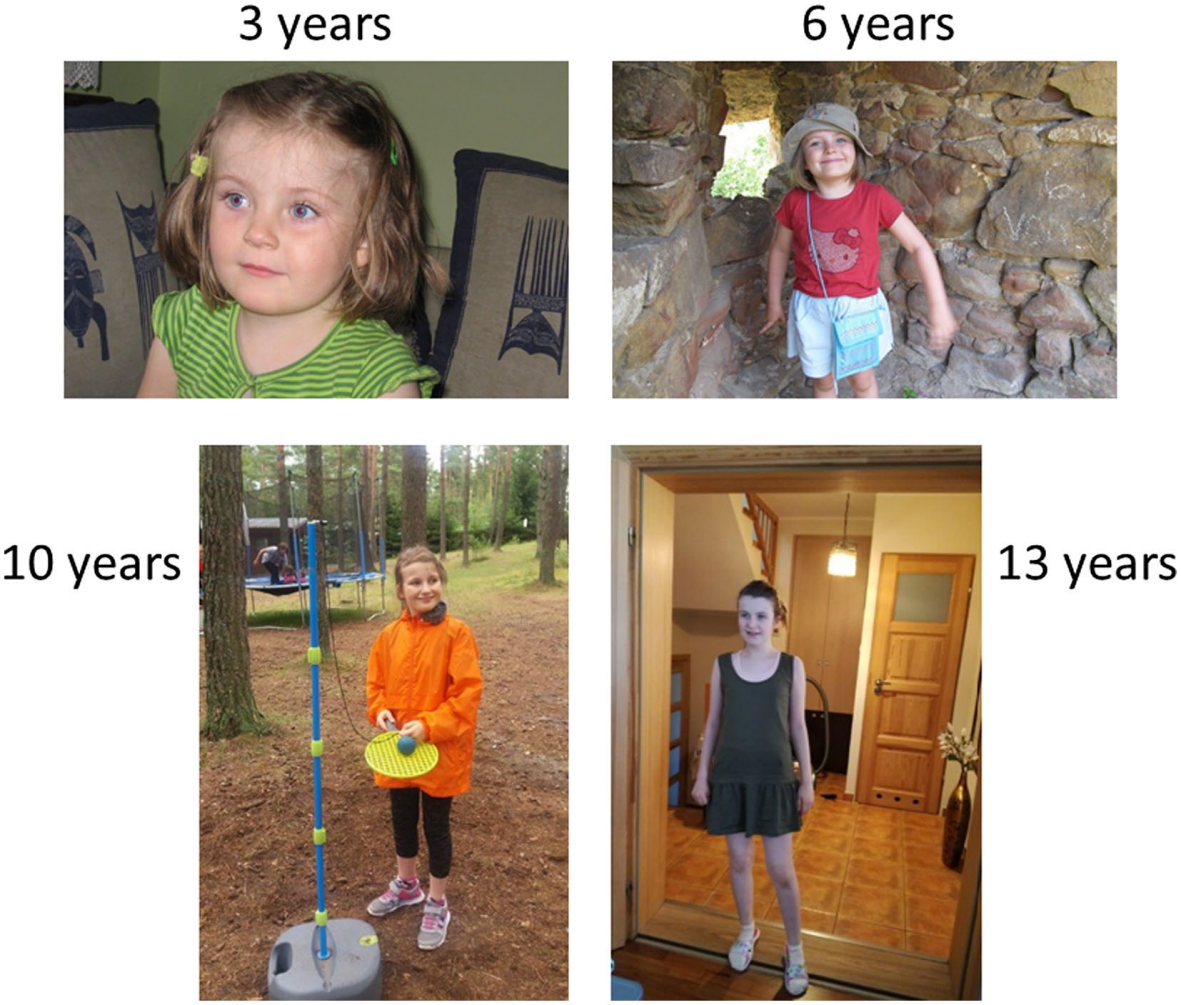
Pictures of patient Z taken at various ages (3, 6, 10, and 13 years, as indicated in the figure). With permission of the parents

Because of psychomotor delay, magnetic resonance imaging was performed, revealing no abnormality in the central nervous system structure (at the age 6 and 11 years). EEG was normal. Organic acids profile in the urine was normal. Intriguingly, no dysmorphology characteristic for MPS III could be observed (Figure 1).

Because of a lack of diagnosis, and progressively (though slowly) developing symptoms, for several years, whole exome sequencing was performed (outsourced by the Department of Medical Genetics of the Warsaw Medical University, Warsaw, Poland, using NGS sequencing with Illumina HiSeq 1500 platform) that revealed occurrence of two mutations in the *NAGLU* gene: c.638C>T and c.889C>T (p.Pro213Leu and p.Arg297Ter). The c.638C>T (p.Pro213Leu) allele was found in the heterozygotic configuration (with the wild‐type allele) in the father, and the c.889C>T (p.Arg297Ter) mutation was detected in the heterozygotic configuration (with the wild‐type allele) in the mother. The c.889C>T (p.Arg297Ter) mutation has been previously reported in the literature and demonstrated to be pathogenic (Yogalingam, Weber, Meehan, Rogers, & Hopwood, 2000), while the c.638C>T (p.Pro213Leu) variant was not described previously, but the location and the kind of the amino acid change suggest it may be pathogenic. Therefore, we conclude that patient Z is a compound heterozygote (c.638C>T/c.889C>T) in the *NAGLU* gene. Metabolic investigation (outsourced by Department of Genetics of the Institute of Psychiatrics and Neurology, Warsaw, Poland) demonstrated slightly increased concentration of heparan sulfate in urine (246 relative to the age‐specific norm of 115 ± 61 mg/g creatinine). Enzymatic assay in serum (outsourced by Department of Genetics of the Institute of Psychiatrics and Neurology, Warsaw, Poland), indicated deficiency of α‐N‐acetylglucosaminidase activity, though about 25% residual enzymatic activity remained (285, relative to the norm of 1,143 ± 103 nmol/ml/42 hr). Measurement of the enzymatic activities in dried blood spot (outsourced by Medical Laboratory Archimed Life Science GmbH, Vienna, Austria) indicated no (i.e., below detection limit) α‐N‐acetylglucosaminidase activity (with control value >0.5 µ mol L^−1^ h^−1^). On the basis of these molecular and biochemical results, the diagnosis of Sanfilippo syndrome B has been made.

At the time of the diagnosis, patient Z was 13 years old. In the clinical examination, 158 cm height (50 percentile), 50 kg weight (60 percentile), and 54 cm head circumference (60 percentile) were measured. Physical examination demonstrated neither facial dimorphism (Figure 1) nor thick or abundant hair. The hepatic and spleen volumes in ultrasound examination were normal. Cardiac ultrasound was normal. Routine laboratory analysis demonstrated no abnormal values. Ranges of motion (ROM) of joints were measured, and the results were as follows (left vs. right): shoulder flexion, 130° versus 140°; shoulder abduction, 120° versus 140°; internal rotation, 70° versus 70°; external rotation, 40° versus 50°; elbow flexion, 150° versus 150°; and full range versus full range for the following parameters: elbow extension; supination (lying down); wrist extension; wrist flexion; hip flexion; hip extension; knee flexion; knee extension; ankle dorsiflexion; ankle plantar flexion. Therefore, ROM were either unchanged or only slightly changed relative to healthy controls. The distance traveled during the 6‐min walk test (6MWT) was 460 meters which was within the age norm.

## 3 CASE DESCRIPTION: PSYCHOLOGICAL ANALYSES

There are different patterns of cognitive functioning in MPS III (Meyer et al., 2007; Valstar, Marchal, Grootenhuis, Colland, & Wijburg, 2011), however, the decline in cognitive functioning and dementia is a symptom that almost always occurs (Valstar, Ruijter, van Diggelen, Poorthuis, & Wijburg, 2008). The psychological examination of patient Z was conducted using various tasks. Firstly, the diagnosis was made with the use of the Stanford‐Binet 5 Intelligence Scale—non‐verbal scale (Roid, 2003). The patient obtained intelligence quotient lower than 40 (IQ < 40). During the examination, the patient presented the abilities of a child at the age of 3 years. The diagnosis with the test was repeatedly disturbed by undesirable behavior, attention problems, and stereotypes, thus, in this case, testing with the SB5 Scale might not provide reliable results. Hence, it was focused on exploring everyday living spheres. More precisely, the situation of diagnosis included clinical trials checking the child's functioning in the scope of everyday activities, as well as elementary school skills (such as writing, grouping elements) and the interview with the parent. The first observation was that she was more willing to work on familiar objects and to cooperate with people she knows. Moreover, she recognized the places she attends regularly and seemed to feel comfortable in them, which is not noticeable in new places (e.g., corridor she had not previously walked). In fact, it was reported that in MPS III patients, it is possible to observe attenuation of behavioral symptoms if they are in familiar places (Escolar et al., 2017).

Patient Z could focus attention for a long time, even an hour. Sometimes short breaks (e.g., a walk in the corridor) after about half an hour of intensive work were necessary. After a break, she easily returned to the task. She had taken her hand away when she felt even a little pressure. The same situation occurred during the greeting, when she immediately took away her hand and looked away. During the walk, the situation was different, as she showed no defenses and did shake her hand instinctively with both mother and a psychologist. She presented defenses against various textures (e.g., cold or rough), while no defense was expressed when painting with hand.

Patient Z did not take objects in her mouth, and did not stimulate her lips. During the meeting with a psychologist, it happened that she put her hands dirty with paint in her mouth or tried to eat plasticine. The behavior manifested as perseverative chewing/hyperorality is frequent in patients with MPS III (Grant et al., 2013), however, in patient Z, it occurred only occasionally.

In the field of motor skills, patient Z was functioning very well. She moved independently, and her gait was steady and stable. The patient freely ran and jumped, climbed the stairs with a delivery step, and entered the objects located on a certain elevation. In the field of fine motor control, she presented great manual skills. She carried out commands in this area willingly. She could pick up even small elements and manipulate them. While performing these tasks, involuntary movements occurred, which prevented her from arranging the tower from blocks. However, placing a small element in a small hole did not cause major difficulties. This is in contrast to the vast majority of MPS III patients in which impairment of motor skills is a frequent symptom (Escolar et al., 2017).

**Figure 2:**
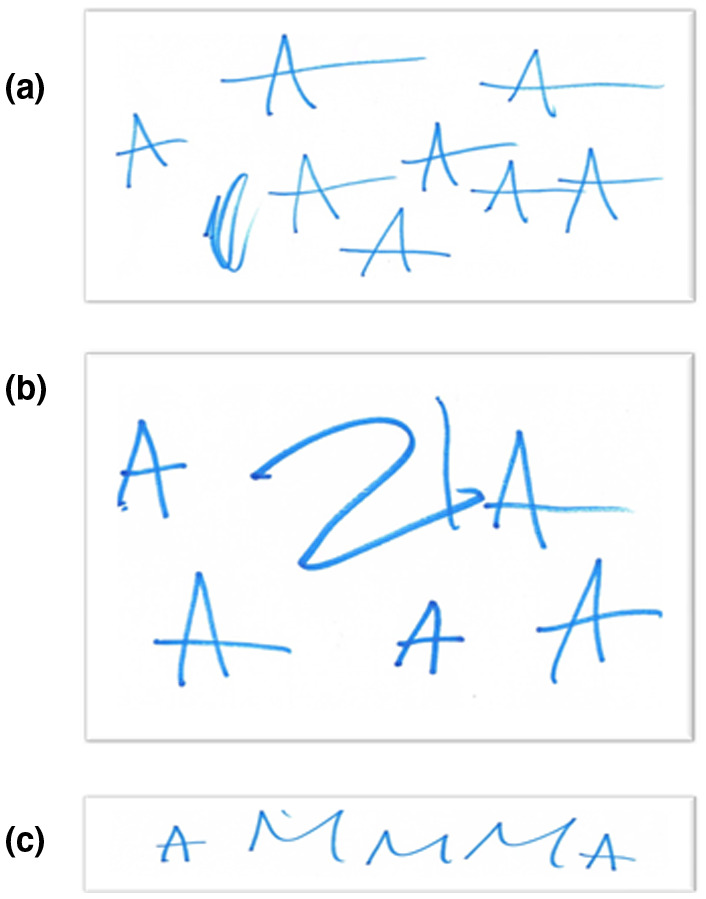
Writing approaches of patient Z, asked to write her diminutive given name “Zuzia” (panel a), asked to rewrite this name (panel b), and asked to write “mama” (panel c)

MPS III patients experience problems with communication and following instructions (including instructions from parents) (Shapiro, Jones, & Escolar, 2017). Nevertheless, when patient Z was asked to write her diminutive name, “Zuzia” (a common Polish female first name), she began to write the letter "A" while starting to repeat "Zuzia" aloud (Figure 2a). The girl preserved the letter and ended when there was not enough space on the sheet of paper and the mother requested to stop. However, when asked to rewrite her name, she has written the letters "ZIA" on a sheet of paper and began to persevere the letter "A" (Figure 2b). When she was asked to write the word MAMA (“mother” in Polish), she has written "AMMMA" (Figure 2c), and she said “MAMA” aloud. The patient perverted some words, including "precisely", "ma'am". She used numbers intentionally—she named individual numbers regardless of the order. Moreover, she used the word "mummy" in reference to his mother, who is the child's main guardian. She noticed when mom was not in the room and asked where she was. She also recognized the emotions “happy” and “sad”, named them and reflected. She did not recognize “fear”, “surprise” or “disgust” or “anger”.

Patient Z could create categories using the criterion of the size, colors of objects, or shapes (also on elements shown in the picture). She matched numeric symbols with the number of objects in a given subset. She could arrange elements in order of size or number of elements in a subset. The correct execution of commands, however, was often disturbed due to numerous perseverations (adding and taking out the same block), as well as the compulsive need to arrange elements in rows. This stereotypic behavior and language are seen in many MPS III patients (Valstar et al., 2011), however, in the case of patient Z, it is less pronounced.

## 4 CONCLUSIONS

Patient Z, who was diagnosed for Sanfilippo syndrome B, is a compound heterozygote (c.638C>T/c.889C>T) in the *NAGLU* gene, with a relatively high (about 25%) residual activity of α‐N‐acetylglucosaminidase. She expresses relatively mild phenotype, with various cognitive and communication functions preserved, and relatively good motoric functions, at the age of 13 years. We suggest that this mild phenotype might arise from partially preserved activity of the p.Pro213Leu variant of α‐N‐acetylglucosaminidase, causing slow accumulation of HS and slow progress of the disease.

## CONFLICT OF INTEREST

The authors declare no conflict of interest.

## AUTHORS’ CONTRIBUTIONS

KP conceptualized the work, analyzed the genetic and biochemical data and drafted the manuscript, AM supervised psychological tests and analyzed their results, ML and JW made clinical observations and provided their description, LG participated in analyses of genetic and biochemical data, PA performed psychological tests and participated in analyses of their results, GW supervised the project, revised the manuscript and prepared its final version.

## INFORMED CONSENT AND ETHICAL COMMITTEE APPROVAL

The study was approved by the local ethics committee, the Medical Ethical Committee of the Medical University of Gdansk, Poland. Written informed consent was obtained from the patient's parents for publication of this case report, including agreement for publication of patient's photographs.

## Data Availability

Original data are available upon request, with respect to the rules of personal data protection.
